# Polymorphic Phases of Supramolecular Liquid Crystal Complexes Laterally Substituted with Chlorine

**DOI:** 10.3390/polym13244292

**Published:** 2021-12-08

**Authors:** Fowzia S. Alamro, Omaima A. Alhaddad, Magdi M. Naoum, Hoda A. Ahmed

**Affiliations:** 1 Department of Chemistry, College of Science, Princess Nourah Bint Abdulrahman University, Riyadh 11671, Saudi Arabia; fsalamro@pnu.edu.sa; 2 Chemistry Department, College of Sciences, Taibah University, Madina Monawara 30002, Saudi Arabia; OHADDAD@taibahu.edu.sa; 3 Department of Chemistry, Faculty of Science, Cairo University, Cairo 12613, Egypt; magdinaoum@yahoo.co.uk; 4 Chemistry Department, College of Sciences, Taibah University, Yanbu 30799, Saudi Arabia

**Keywords:** polymorphic phases, lateral chloro, supramolecular dimers, hydrogen bonding, induced phase, mesomorphic properties

## Abstract

New supramolecular complexes, based on H-bonding interactions between 4-(pyridin-4-yl) azo-(2-chlorophenyl) 4-alkoxybenzoates (**B***n*) and 4-[(4-(n-hexyloxy)phenylimino)methyl]benzoic acid (**A***6*), were prepared and their thermal and mesomorphic properties investigated via differential scanning calorimetry (DSC) and Fourier-transform infrared spectroscopy (FT-IR) in order to confirm their H-bonding interactions. The mesophase behavior of each mixture was examined by DSC and polarized optical microscopy (POM). According to the findings of the study, in all of the designed mixtures, the introduction of laterally polar chlorine atom to the supramolecular complexes produces polymorphic compounds possessing smectic A, smectic C and nematic mesophases, in addition, all products have low melting transitions. Thermal stabilities of the associated phases depend on the position and orientation of the lateral polar Cl^−^ atom as well as the length of terminal flexible alkoxy chain. Comparisons were made between the present lateral Cl^−^ complexes and previously investigated laterally-neat complexes in order to investigate the impact of the addition, nature and orientation of polar substituent on the mesomorphic behavior. The investigations revealed that, the polarity and mesomeric nature of inserted lateral substituent into the base component play an essential role in affecting their mesomorphic properties. Furthermore, for current complexes, induced polymorphic phases have been found by introducing the chlorine atom.

## 1. Introduction

Supramolecular hydrogen bonded complexes (SMHBCs) have recently attracted an increasing amount of scientific attention [1,2,3,4,5]. Supramolecular chemistry [6] and liquid-crystals (LCs) [7,8] in these systems, proved to have efficient qualities for optical and industrial applications [9,10]. H-bonding intermolecular interactions [1,2,3,4,5] are a good approach for designing self-assembly LCs via multiple non-covalent bonds. Several investigations indicated that the combination of a carboxylic acid and a pyridine derivative, in a hydrogen-bonded complex, are the optimum H-bond acceptor and donor and the best choice for most of these studies. Furthermore, the use of multifunctional components in the development of non-covalent interactions can result in improved characteristics of SMHB LC network architectures [6,7].

Because of their abilities to cis/trans isomerization, as a result of temperature and light irradiation, azo-pyridine molecules are incorporated within the liquid-crystal molecule to make them photo-responsive [6,7]. Modifying the core structure of azo-pyridine based derivatives or adding lateral substituents might result in significant changes in their photo-physical and photochemical capabilities [6,7]. Many properties of liquid crystalline materials are greatly improved by the addition of lateral groups of varying size and polarity. This could be due to the disruption of molecular packing, which lowers both the melting temperature and thermal stability of LC mesophases [11,12,13,14,15,16,17,18]. Azo-pyridines have recently been employed to create nano-fiber supramolecular self-assembling and hydrogen/halogen-bonding LCs with photo-induced transition phenomena [19,20,21,22,23].

Our research focuses on designing photosensitive SMLCs by intermolecular interactions with the appropriate H-bond donors and acceptors [24,25,26,27,28,29,30,31,32,33]. The overall molecular architectures, as well as the combination of rigid (aromatic) and flexible segments (alkyl chains), produce anisotropic structures. These changes in LC features may have an impact on mesomorphism as well as the properties important for their technical applications [34,35,36,37].

Several two- or three-ring systems based on Schiff base LCs have also been reported and their mesomorphic properties investigated in order to investigate the correlation between the geometry of mesogens and their mesomorphic properties [38,39,40,41,42]. In general, the mesomorphic thermal stability depends on the polarity and/or polarizability of the different mesogens present within the molecule. Most of the investigated derivatives for the formation of LC blends, through H-bonding interactions, are derivatives of benzoic acid [43,44,45].

**Figure 1:**
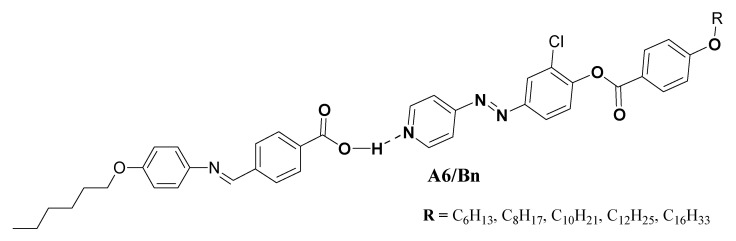
Supramolecular complexs, **A***6*/**B***n*.

The aim of the present investigation is to construct novel H-bonded supramolecular complexes of new conformation (Figure 1), between 4-(pyridin-4-yl) azo-(2-chlorophenyl) 4-alkoxybenzoates (**B***n*) and 4-[(4-(n-hexyloxy)phenyl iminomethine]benzoic acid (**A***6*), in order to explore the geometrical and thermal parameters of the examined complexes to better understand and regulate the mesomorphic features of soft material complexes. Moreover, the effect of different spatially oriented lateral-polar groups on the thermal and phase behavior of produced intermolecular H-bonded complexes with varying positions on the central ring of the azo-pyridine based moiety was investigated.

## 2. Experimental

### 2.1. Preparation of Supramolecular Complexes

The two components 4-[(4-(n-hexyloxy)phenylimino)methine]benzoic Acid (**A***6*), and lateral chloro azo-pyridine derivatives (**B***n*), were tested to exhibit identical transition temperatures as previous investigations [45,46].

**Scheme 1:**
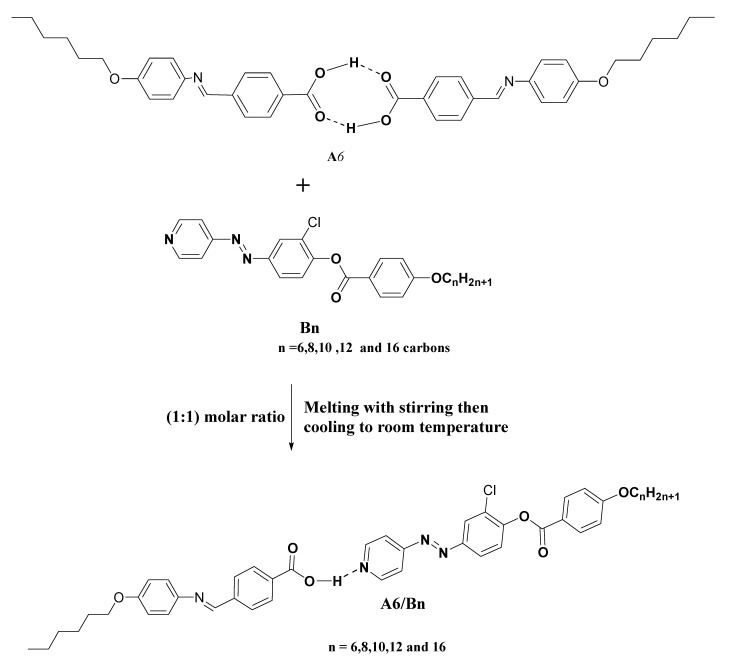
Preparation steps of 1:1 SMHBCs (**A***6*/**B***n*).

The 1:1 molar ratios of any two complementary components SMHBCs (**A***6*/**B***n*) were made by melting the required amounts of each component at approximately 230 °C, stirring to achieve an intimate blend, and then cooling to room-temperature with stirring. (Scheme 1). An example to prepare the SMHBC **A***6*/**B***10*: 0.0325 mg of 4-[(4-(n-hexyloxy)phenylimino)methyl]benzoic Acid (**A***6*) and 0.0494 mg of 4-(2-(pyridin-4-yl)diazenyl-(2-chlorophenyl) 4-decyloxy benzoate (**B***10*) were melted together to form the complex.

### 2.2. Characterizations

Details are given in supplementary data and Figures S1 and S2.

A diamond tip Perkin Elmer ATR spectrometer was used to record FT-IR spectra for structural characterization. Fourier transform infrared spectroscopy was used to record all spectra with wave numbers between 4000 and 400 cm^−1^. All spectra were obtained at room temperature and vector normalized after being calibrated for the base line. The number of scan and spectral resolution were 32 scan and 4 cm^−1^; respectively.

## 3. Results and Discussion

### 3.1. Spectroscopic Confirmation of SMHBCs Interactions

**Figure 2:**
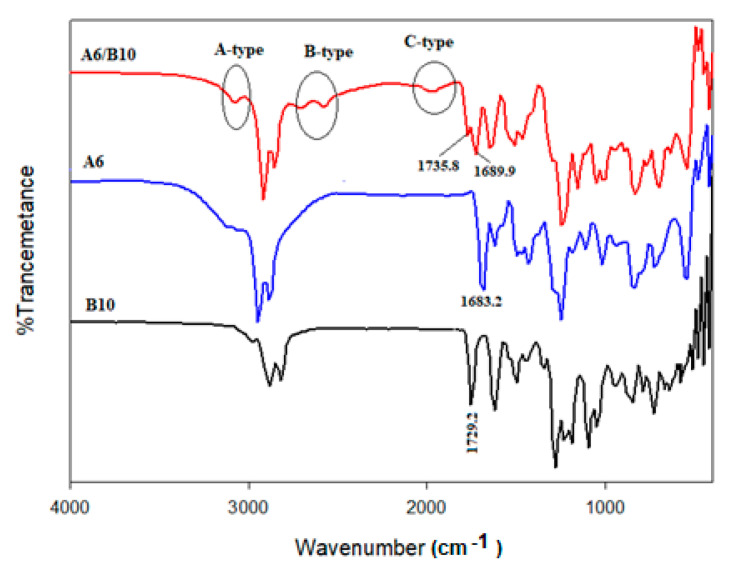
Collective FT-IR spectra of acid **A***6*, base **B***10* and their complex **A***6*/**B***10*.

FT-IR spectra data have demonstrated the formation of SMHBCs. Individual derivatives as well as their H-bonded supramolecular complexes were subjected to FT-IR spectroscopic measurements. Figure 2 shows, as examples of the FT-IR spectrum of acid **A***6*, azo-pyridine base **B***10*, and their complex **A***6*/**B***10*. It was reported that, there was no substantial effect of the length of the alkoxy chain on the wave number of the C=O group stretching vibration for the azo-pyridine homologues [46,47]. As seen from Figure 2, the stretching vibration of the C=O group of the hexyloxy acid derivative (**A***6*) was assigned to the signal at ≈1683 cm^−1^. The stretching vibration of the C=O carboxylic moiety is one of the most important indicators for the interactions of H-bonded supramolecular complexes. The strength of the O-H bond is reduced when the carboxylic group OH-group is shared in the H-bonding formation. The FT-IR data indicated that the formation of H-bonds had substantial influence on the stretching frequency of the C=O group of the free carboxylic acid (nearly 7 cm^−1^). The supramolecular complex formation, on the other hand, has a strong stretching vibration effect on the C=O of the azo-pyridine base’s ester bond, with their wave number increasing from 1729 to 1736 cm^−1^ for complex **A***6*/**B***10*. That attributed to the polarity of electron-withdrawing Cl-atom enhanced the double bond character of ester linkage in azo-pyridine component and leads to an increment in the absorption of carbonyl group. Furthermore, it has been observed [48,49,50,51,52] that the presence of three Fermi resonance vibration bands of the H-bonded OH groups A-, B-, and C-types is a substantial evidence for the formation of H-bond within the complexes. The vibrational peak of the A-type Fermi band of SMHBC **A***6*/**B***10* emerges at ≈3040 cm^−1^. In addition, the peak at ≈2543 cm^−1^ could be due to both the O-H in-plane bending vibration and its fundamental stretch (B-type). The C-type Fermi band was allocated at ≈1925 cm^−1^ due to the interaction between the overtone of the torsional effect and the basic stretching vibration of the OH.

### 3.2. Mesomorphic Study

**Figure 3:**
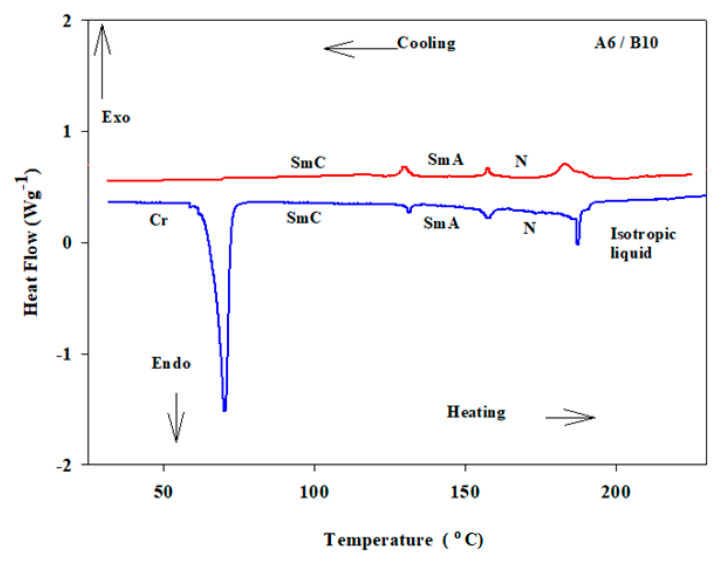
DSC thermogram of **A***6*/**B***10* SMHBC at heating rate 10 °C min^−1^ during heating and cooling scans.

All designed complexes (1:1 molar ratio), **A***6*/**B***n*, were prepared from one homologue of the acid component (**A***6*), and five homologues of the azo-pyridine component (**B***n*). The characterization for their mesophase properties was carried out by POM and DSC. The mesomphase textures formed under the POM were verified by the DSC analyses and types of phases were identified for all investigated complexes (**A***6*/**B***n*). An example of DSC thermogram is depicted in Figure 3 for the 1:1 **A***6*/**B***10* SMHBC. DSC transitions were measured for second heating/cooling cycles to ensure the thermal stability of the designed complexes.

**Figure 4:**
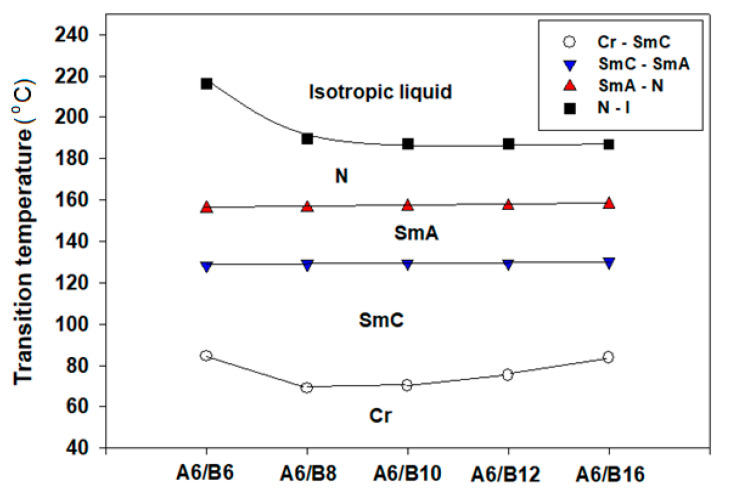
Effect of the terminal-chain length (*n*) on the phase behavior of the 1:1 SMHBCs, **A***6*/**B***n*.

Mesomorphic transitions (temperatures, enthalpies and entropies) values were analyzed by DSC for all formed SMHBCs and are collected in Table 1. The attached terminal flexible chain length (*n*) of the azo-pyridine base component is associated with high affect on the produced properties of 1:1 mixtures so its impact is represented graphically in Figure 4. The data in Table 1 and Figure 4 indicate that, independent of terminal carbon chain length (*n*) for base derivative, all prepared SMHBCs exhibit enantiotropic polymorphic phases. The N phases are observed to cover all complexes in addition to the smectic phases (SmC and SmA). As usual, the N thermal stability (***T***_N-I_) was found to decrease with length of terminal chain, *n*.

It should be noted that, the present azo-pyridine homologous series (the lateral Cl atom attached to the meta-position with respect to the ester carbonyl linkage) is purely nematogenic possessing the N phases with low thermal stabilities [46] while the 4-[(4-(n-hexyloxy)phenylimino)methine]benzoic acid [43] exhibits dimorphic transitions of SmC and N mesophases with high thermal stability. We can deduce from Table 1 and Figure 4 that, all formed complexes **A***6*/**B***n* exhibit an induced SmA mesophase independent of the terminal length of chains (*n*). Moreover, the smectic phases stabilities are slightly enhanced with the increment of *n* (Figure 4). The insertion of the lateral Cl atom in SMHBC structure weakens the side-by-side cohesion forces, thus increasing the predominance of the N phase formation for all 1:1 SMHBCs compared by our previous laterally neat SMHBCs [53], that briefly discussed in Section 3.3. Moreover, the molecular architecture and volume of the lateral moiety affect the mesophase thermal stability and the polarizability of whole molecule [35]. Further, the length of the terminal chain and the orientation of the lateral substituent are essential factors in determining the type and the temperature range of the produced mesophase.

**Figure 5:**
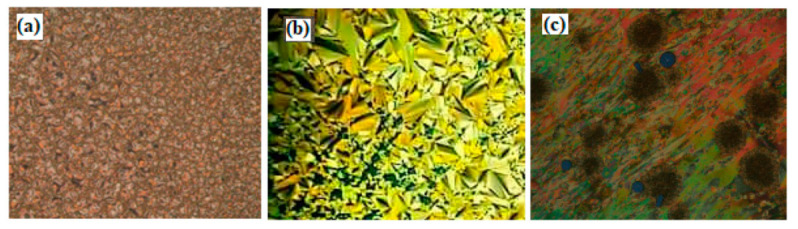
Textures under POM of the polymorphic phases for **A***6*/**B***10* SMHBC; (**a**) SmC phase at 110.0 °C; (**b**) SmA phase at 149.0 °C; (**c**) N phase at 180.0 °C.

As can be seen from Table 1 and Figure 4, the mesomorphic ranges of the investigated SMHBCs **A***6*/**B***n* decrease with *n*. The complex **A***6*/**B***6* exhibits the highest mesomorphic temperature range (131.6 °C) than the other complexes, whereas, **A***6*/**B***16* has relatively the smallest mesophase range (103.2 °C). The wide N temperature rang value is also confirmed for **A***6*/**B***6* enantiotropically. Moreover, the N stability decreases with the alkoxy chain length of the base component. Additionally, the melting transitions of present SMHBCs are slightly affected by the length of the terminal chain (*n*) of the azo-pyridines. From the present investigation, it could be concluded that, as the molecular anisotropy increases as a result of lengthening of the acid moiety and due to the position of the lateral Cl atom in the SMHB structural shape, the stability of mesophases are increased and an induced SmA phase is produced compared to previous laterally-neat series [53]. Moreover, it was found that from computational calculations of azo-pyridines [45], the introduction of Cl lateral substituent increases the polarizability and shows enhancement of mesophase stability temperature range of whole molecule. Textures of the formed mesophases as examples from POM are shown in Figure 5.

### 3.3. Effect of Position and Role of Lateral Polar Group on the SMHBCs Stability

**Figure 6:**
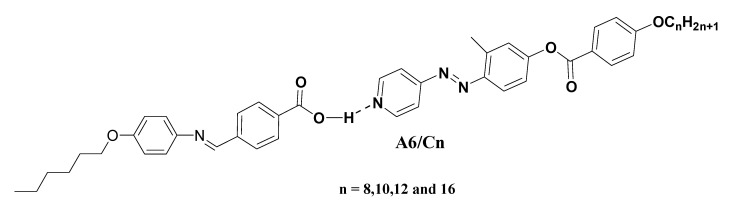
Supramolecular complexs, **A***6*/**C***n*.

**Figure 7:**
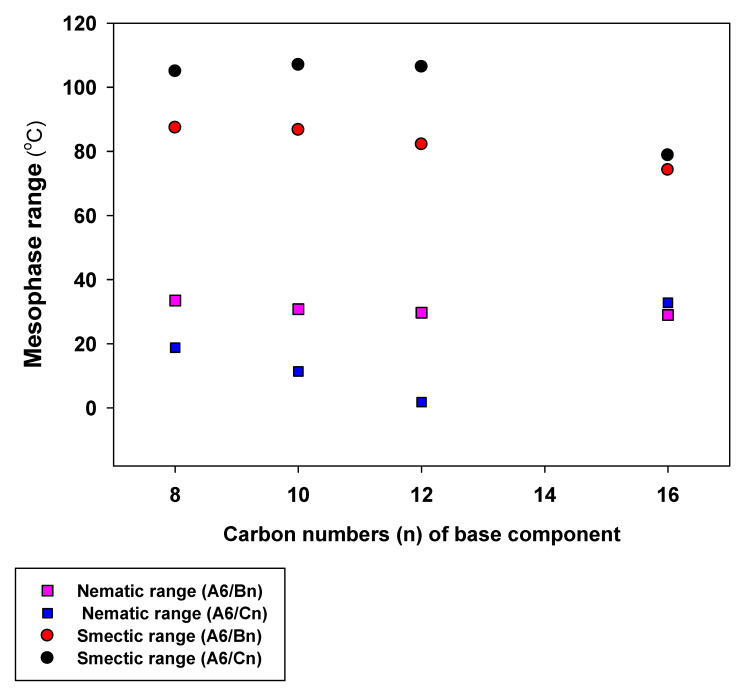
Dependence of the mesophase temperature range on the length of the terminal alkoxy-chain (*n*) of the two analogous complexes **A***6*/**B***n* and **A***6*/**C***n*.

The comparison between the mesomorphic behavior of present investigated SMHBCs (**A***6*/**B***n*) and their corresponding laterally substituted CH_3_ analogues, attached in meta-position with respect to the carbonyl ester group SMHBCs (**A***6*/**C***n*, Figure 6) [40] was conducted to analyze the effect of position (spatial orientation) and polarity of the lateral substituent on the mesophase transition stabilities of the two type of 1:1 supramolecular complexes (**A***6*/**B***n* and **A***6*/**C***n*). Values of thermal stabilities of both series were illustrated graphically in Figure 7. The designed SMHBCs exhibit polymorphic phases covering all alkoxy terminal chains. The smectic and nematic ranges vary linearly with all length of alkoxy-terminal chain (*n*) (Figure 7). On the other hand, the nematic temperature ranges of **A***6*/**B***n* complexes are broader than those of **A***6*/**C***n* SMHBCs analogues. On the other side, the smectogenic temperature ranges of **A***6*/**C***n* complexes showed wide ranges than **A***6*/**B***n* complexes. That is, the strength of the intermolecular interactions between molecules strongly affects both the stability and temperature ranges of the mesophase formed. Additionally, the location and the mesomeric nature of the lateral group attached to the base component of the complex greatly affects the polarizability of the H-donors and acceptors and thus impacts their strength of the hydrogen bonding interactions [54]. Furthermore, the location of lateral electron withdrawing Cl-moiety in the ortho-position with respect to ester group increases the polarity of whole molecule and consequently enhances the end-to-end interaction to influence the nematic (N) phase [55], thus resulting in increased N stability and producing a wide temperature range of the N phase that appears in all homologues of azo-pyridines (**B***n*).

**Figure 8:**
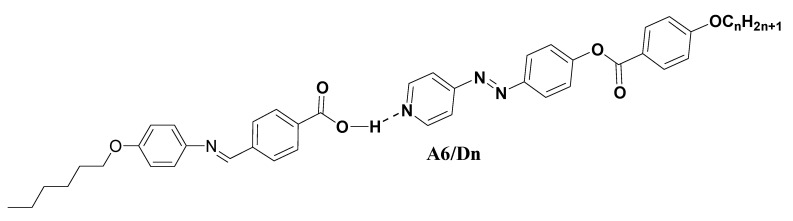
Supramolecular complexs, **A***6*/**D***n*.

The purpose of this study was seeing what effect introducing the lateral polar Cl atom on the mesomorphic behavior of 1:1 molar mixtures of SMHBCs (**A***6*/**B***n*), another comparison was established between the mesophase stabilities and types of the current complexes and the previously laterally-free SMHBCs analogues (**A***6*/**D***n*, Figure 8) [53]. The study revealed that the laterally-neat complexes (**A***6*/**D***n*) possess purely SmA mesophase with high thermal stabilities. Thus, the addition of lateral Cl atom in the ortho position with respect to the ester spacer induces polymorphic phases (SmC, SmA and N phases). Moreover, the polarity of the electron withdrawing Cl moiety enhances the nematic phases’ stabilities and mesomorphic temperature ranges.

### 3.4. Entropy Change of SMHB Complexes

**Figure 9:**
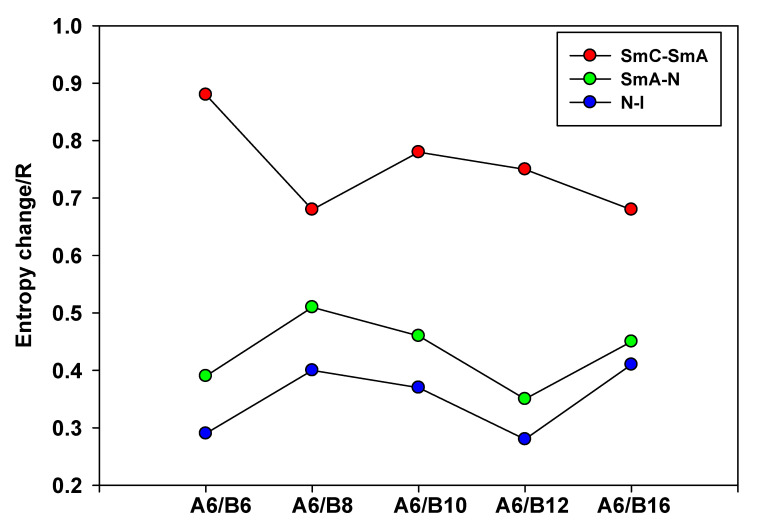
Entropy changes for SMHBCs **A***6*/**B***n*.

Normalized entropy changes (***∆S***/R) for the present SMHBCs **A***6*/**B***n* of all mesomorphic transitions were constructed in Figure 9. For each mesophase transition Figure 9 shows that, independent of the number of carbons in terminal chains, irregular values of entropy changes were observed. Such irregular trends may be attributed to the intermolecular interactions that depend on both the position and rotation of the lateral electron-withdrawing Cl substituent which accordingly affect the molecular ordering of whole molecule of the complex [56,57]. The resulted lower magnitude of entropy changes for the nematic transitions of homologous complexes may be explained in terms of the lower degree of the linear alignments of the molecules at high temperatures, whereas the degree of alignments are highly increased at lower temperature within the smectic mesophases. Furthermore, the terminal chains have pronounced role to make multi-conformational changes in the molecule. The increment in the conformation and orientation changes of the whole SMHBCs are in accordance with the estimated entropy changes.

## 4. Conclusions

Based on laterally Cl azo-pyridine derivatives and 4-[(4-(n-hexyloxy)phenyliminomethine]benzoic acid, five new polymorphic complexes of 1:1 SMHB liquid crystalline complexes were prepared. DSC, POM, and FT-IR measurements confirmed the structure of all of the formed complexes. All of the 1:1 molar H-bonded complexes investigated were shown to be polymorphic and processing low melting temperatures. The increased stability of the laterally chloro-substituted homologues was attributed to their high degree of molecular interaction, as a result of increasing the polarity of the complex molecule, which allows for more complex packing than that of their methyl-substituted analogues. Moreover, induced polymorphic phases have been produced after introduction of the chlorine atom in the geometrical skeleton. It can be inferred that the development of new nematogenic supramolecular H-bonded conformers with specified molecular geometry represents a possible approach for achieving proper mesophase properties. Furthermore, the increases in conformational and orientation changes of the whole SMHBCs are consistent with the estimated entropy changes.

## Figures and Tables

**Table 1:** Phase transition temperatures, °C (enthalpy of transitions, kJ/mol), total mesophase temperature range and normalized entropy change for the SMHBCs, **A***6*/**B***n*.

System	*T*_Cr-SmC_	*T*_SmC-SmA_	*T*_SmA-N_	*T*_N-I_	*∆T*	*∆S/R*_C-A_	*∆S/R*_A-N_	*∆S/R*_N-I_
**A***6*/**B***6*	84.5 (35.30)	128.3 (2.93)	155.9 (1.38)	216.1 (1.16)	131.6	0.88	0.39	0.29
**A***6*/**B***8*	68.9 (29.83)	129.1 (2.29)	156.3 (1.83)	189.8 (1.54)	120.9	0.68	0.51	0.40
**A***6*/**B***10*	70.3 (52.93)	129.3 (2.61)	157.0 (1.65)	187.4 (1.42)	117.1	0.78	0.46	0.37
**A***6*/**B***12*	75.1 (49.52)	129.5 (2.52)	157.3 (1.27)	187.0 (1.09)	111.9	0.75	0.35	0.28
**A***6*/**B***16*	83.7 (40.06)	130.1 (2.29)	157.9 (1.63)	186.9 (1.58)	103.2	0.68	0.45	0.41

Abbreviations: Cr = crystal; N = nematic; SmC = smectic C; SmA = smectic A; I = isotropic liquid.

## Data Availability

The data presented in this study are available on request from the corresponding author.

## Supplementary Materials

The following are available online at https://www.mdpi.com/article/10.3390/polym13244292/s1, Scheme S1. Synthesis of 4-[(4-(n-hexyloxy)phenylimino)methyl]benzoic Acid (**A***6*); Figure S1. ^1^H NMR of **A***6*; Figure S2: C13 NMR of A6; Figure S3. DSC thermogram of **A***6*/**B***6* SMHBC at heating rate 10 °C min^−1^ during heating and cooling scans; Figure S4. DSC thermogram of **A***6*/**B***8* SMHBC at heating rate 10 °C min^−1^ during heating and cooling scans; Figure S5. DSC thermogram of **A***6*/**B***12* SMHBC at heating rate 10 °C min^−1^ during heating and cooling scans; Figure S6. DSC thermogram of **A***6*/**B***16* SMHBC at heating rate 10 °C min^−1^ during heating and cooling scans.

## Sample Availability

Samples of the compounds are available from the authors.
