# Supplementary material for: Polymorphic Phases of Supramolecular Liquid Crystal Complexes Laterally Substituted with Chlorine
Source: Polymers (Basel). 2021 Dec 8;13(24):4292. doi: 10.3390/polym13244292 (PMC8703890; doi:10.3390/polym13244292)
Supplement: Supplementary file 1 [file polymers-13-04292-s001.zip › polymers-1480835-supplementary.pdf]

# Supplementary Data

# Polymorphic phases of supramolecular liquid crystal complexes laterally substituted with chlorine

**Fowzia S. Alamro [1], Omaima A. Alhaddad [2] , Magdi M. Naoum [3] and Hoda A. Ahmed [3,4,*]**

1 Department of Chemistry, College of Science, Princess Nourah bint Abdulrahman University, Riyadh 11671, Saudi Arabia; fsalamro@pnu.edu.sa
2 Chemistry Department, College of Sciences, Taibah University, Madina Monawara 30002, Saudi Arabia; alhaddad105@yahoo.com
3 Department of Chemistry, Faculty of Science, Cairo University, Cairo 12613, Egypt; magdinaoum@yahoo.co.uk
4 Chemistry Department, College of Sciences, Yanbu, Taibah University, Yanbu 30799, Saudi Arabia

* Correspondence: (H.A.A.) ahoda@sci.cu.edu.eg

## 1. Materials

4-Hexyloxyaniline was purchased from Sigma Aldrich (Germany). 2-Chorophenol was obtained from Merck (Germany). N,N′-Dicyclohexylcarbodiimide (DCC), 4-aminopyridine, 4-formylbenzoic acid and 4-dimethylaminopyridine (DMAP) were purchased from Aldrich (Wisconsin, USA). All solvents of pure grade were used and are purchased from Aldrich.

## *2. Synthesis*

Acid **A6** were prepared according to the following scheme:

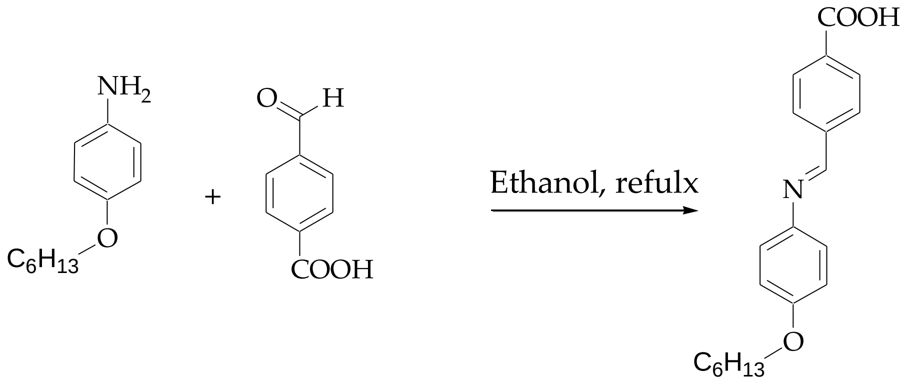


**A6**

**Scheme S1:**Synthesis of 4-[(4-(n-hexyloxy)phenylimino)methyl]benzoic Acid (**A***6*).

Equimolar amount of 4-formylbenzoic acid (4.0 mmol) and 4-n-hexyloxyaniline (4.0 mmol) in ethanol (10 ml) were refluxed for two hours. The reaction mixture was allowed to cool and the separated product was filtered. The obtained solid was recrystallized from ethanol.

Yield: 92.0 %; mp 190.0 ˚C, FTIR (ύ, $cm^{-1}$): 2930-2864 ($CH_2$ stretching), 1678 (C=O), 1616 (C=N),1597 (C=C), 1490 (C–O $_{Asym}$), 1235 (C-O $_{Sym}$). $^1$H NMR (400 MHz, $CDCl_3$) δ 10.03 (s, 1H, CH=N), 8.13 (d, *J* = 8.5 Hz, 2H, ArH), 7.97 (d, *J* = 8.5 Hz, 2H, ArH), 7.40 (d, *J* = 8.4 Hz, 2H, ArH), 6.99 (d, *J* = 8.6 Hz, 2H, ArH), 4.05 (t, *J* = 6.5 Hz, 2H, $CH_3(CH_2)_3CH_2\underline{CH_2}$), 1.91 – 1.76 (m, 2H, $CH_3(CH_2)_3\underline{CH_2}CH_2$), 1.58 – 1.56 (m, 6H, $CH_3\underline{(CH_2)_3}CH_2CH_2$), 0.88 (t, *J* = 6.6 Hz, 3H, $\underline{CH_3}(CH_2)_3CH_2CH_2$). $^{13}$C NMR (101 MHz, $CDCl_3$) δ 191.62, 169.63, 159.57, 139.62, 134.29, 130.78, 129.60, 124.37, 115.44, 115.42, 68.39, 31.55, 29.22, 25.64, 22.60, 14.05. Elemental analyses: Found (Calc.): C, 73.79 (73.82); H, 7.11 (7.12); N, 4.29 (4.30).

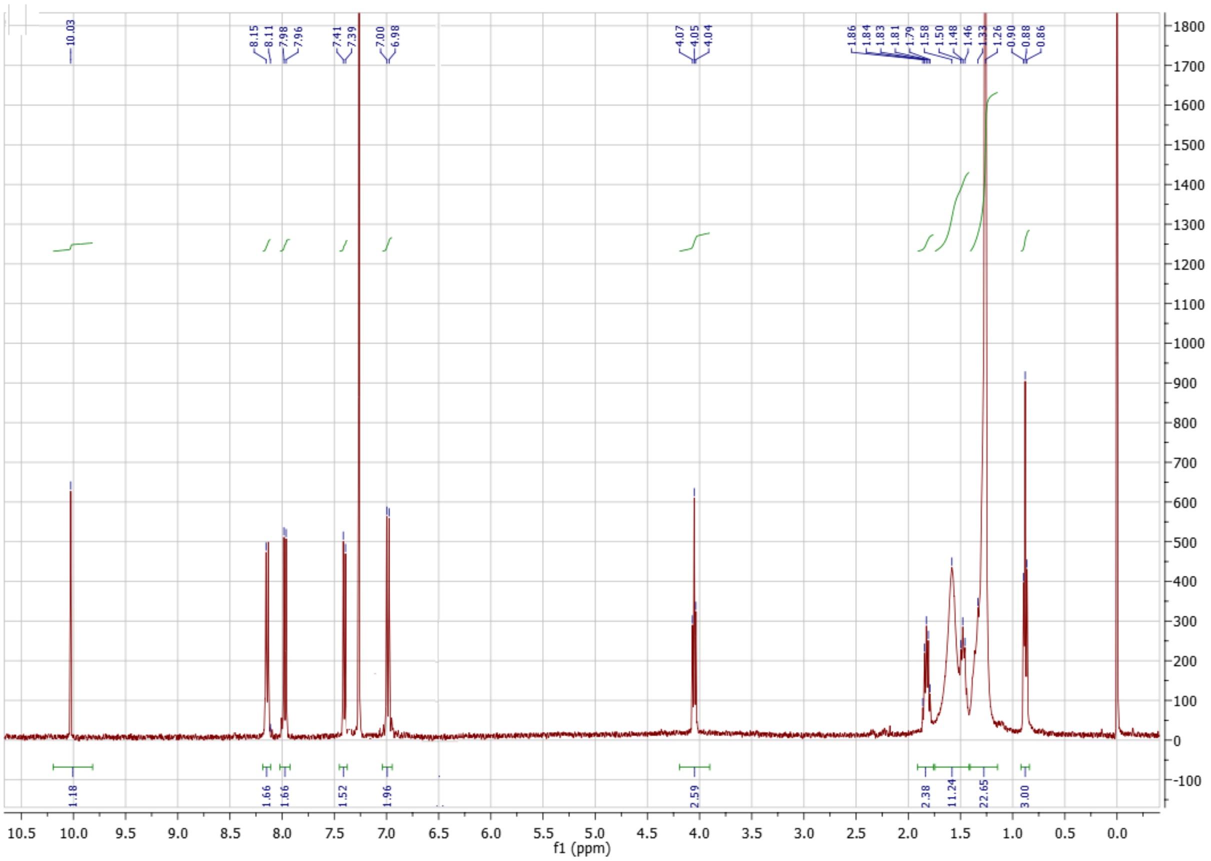


**Figure S1: $^1$H NMR of A6**

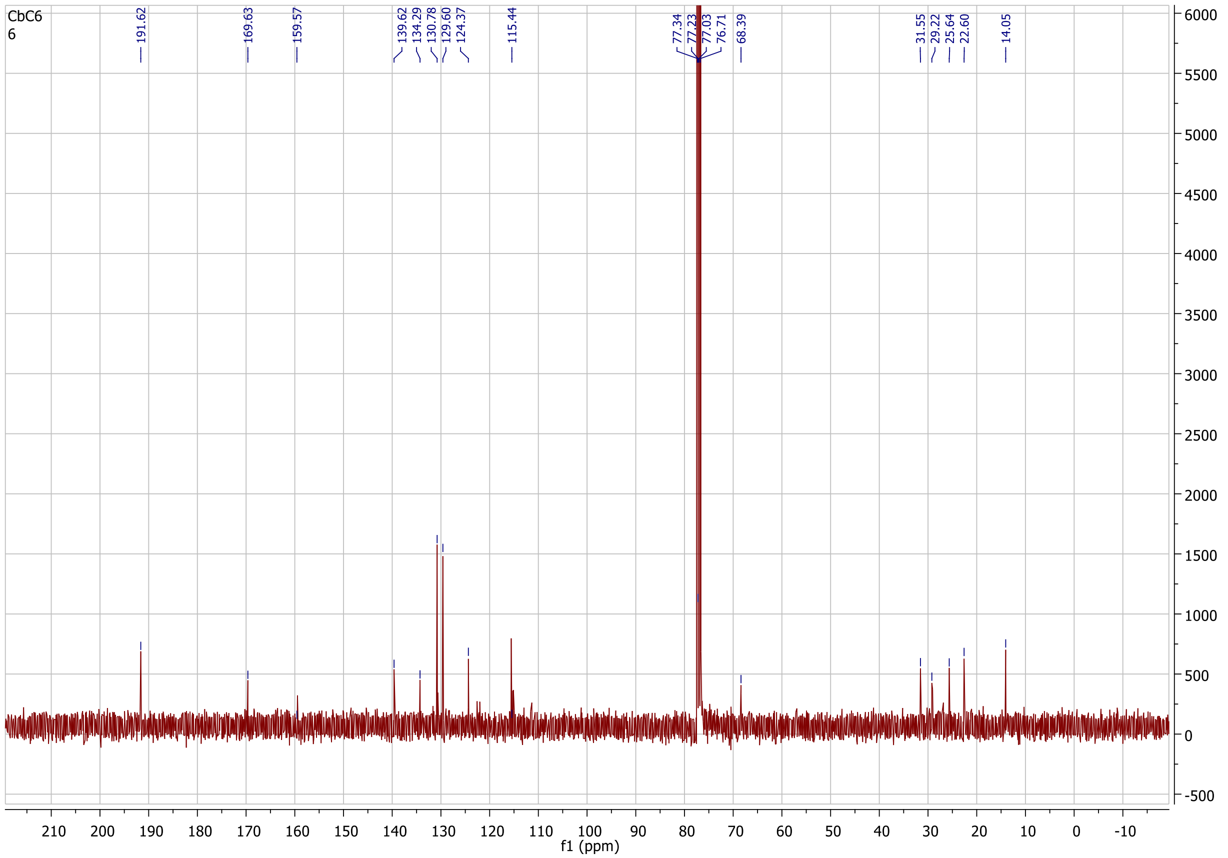


**Figure S2: C[13] NMR of A6**

***Synthesis of 4-(2-(pyridin-4-yl)diazenyl-2- chlorophenol***

A cold solution of (3.5 g, 27 mmol) 2-chorophenol in 10% sodium hydroxide (50 ml) was added with stirring to a cold solution of sodium nitrite (2.0 g, 30 mmol) in water (20 ml). The resulting solution was added to a cold solution of 4-aminopyridine (2.5 g, 27 mmol) in 6 molar HCl with stirring for 10 min keeping the temperature below 5°C. Subsequently, to complete precipitation, the reaction mixture was neutralised by sodium carbonate till pH ≈ 6. A formed orange dye was recrystallised from hot aqueous ethanol (1:1).

**Synthesis of 4-(pyridin-4-yl) azo-(2-chlorophenyl) 4-alkoxybenzoates, B*n***

A mixture of 4-(2-(pyridin-4-yl)diazenyl-(2-chlorophenol (4.1 mmol) and 4-alkoxybenzoic acid (4.1 mmol) was dissolved in dry methylene chloride (25 ml). DCC (0.02 mol) and few crystals of DMAP, as catalyst, were added. The mixture kept under stirring for 72 h at room temperature. The solid residue obtained filtered and the filtrate was evaporated till dryness and the solid was recrystallised twice from ethanol.

**4-(2-(Pyridin-4-yl) azo-(2-chlorophenyl) 4-decyloxybenzoate, B*10***

Yield: 94.9%; mp 72.8°C, FTIR (ύ, $cm^{-1}$): 2921- 2857 ($CH_2$ stretching), 1737 (C=O), 1600 (C=C), 1471 (C–O Asym), 1410 (N=N), 1225 (C–O Sym). 1H NMR (400 MHz, $CDCl_3$): δ/ppm: 0.82 (t, 3H, $CH_3(CH_2)_7CH_2$ $CH_2$, J = 6.6 Hz), 1.21–1.41 (m, 14H, $CH_3(CH_2)_7$ $CH_2$ $CH_2$), 1.73–1.81 (m, 2H, $CH_3(CH_2)_7CH_2CH_2$), 3.98 (m, 2H, $CH_3(CH_2)_7CH_2CH_2$), 6.92 (m, 2.35 H, Ar–H), 7.19 (s, 1H, Ar–H, E-isomer), 7.43–7.41 (m, 2.10 H, Ar–H), 7.89 (d, 2H, J = 8.2 Hz, Ar–H E-isomer), 8.02–8.12 (m, 3.20 H, Ar–H), 8.2 (d, 0,12 H, J = 8.4 Hz, Ar–H Z-isomer),), 8.4 (d, 0.12 H, J = 8.3 Hz, Ar–H, Z-isomer), 8.7 (d, 1 H, J = 8.5 Hz, Ar–H, E-isomer). 13C NMR (101 MHz, CDCl3) δ:ppm: 164.00, 163.72, 151.76, 150.37, 149.88, 147.64, 147.21, 132.66, 128.39, 127.38, 124.50, 124.20, 123.80, 120.40, 114.50, 68.42, 31.91, 29.67, 29.19, 29.08, 29.04, 25.99, 22.70, 15.30, 14.15. Elemental analyses: Found (Calc.): C, 68.04 (68.07); H, 6.50 (6.53); N, 8.49 (8.51); Cl, 7.16 (7.18).

**4-(2-(Pyridin-4-yl) azo-(2-chlorophenyl) 4-dodecyloxybenzoate, B*12***

Yield: 91.2%; mp 75.2°C, FTIR (ύ, $cm^{-1}$): 2921- 2857 ($CH_2$ stretching), 1735 (C=O), 1600 (C=C), 1470 (C–O Asym), 1410 (N=N), 1224 (C–O Sym). 1H NMR (400 MHz, $CDCl_3$): δ/ppm: 0.82 (t, 3H, $CH_3(CH_2)_7CH_2$ $CH_2$, J = 6.6 Hz), 1.21–1.41 (m, 14H, $CH_3(CH_2)_7$ $CH_2$ $CH_2$), 1.73–1.81 (m, 2H, $CH_3(CH_2)_7CH_2CH_2$), 3.94 (m, 2H, $CH_3(CH_2)_7CH_2CH_2$), 6.93 (m, 2.34 H, Ar–H), 7.19 (s, 1H, Ar–H, E-isomer), 7.42–7.40 (m, 2.10 H, Ar–H), 7.89 (d, 2H, J = 8.2 Hz, Ar–H E-isomer), 8.03–8.12 (m, 3.20 H, Ar–H), 8.2 (d, 0,12 H, J = 8.4 Hz, Ar–H Z-isomer),), 8.4 (d, 0.12 H, J = 8.3 Hz, Ar–H, Z-isomer), 8.8 (d, 1 H, J = 8.5 Hz, Ar–H, E-

isomer). Elemental analyses: Found (Calc.): C, 69.01 (69.02); H, 6.93 (6.95); N, 8.02 (8.05); Cl, 6.76 (6.79).

**4-(2-(Pyridin-4-yl) azo-(2-chlorophenyl) 4-hexadecyloxybenzoate, B*16***

Yield: 92.0%; mp 85.0°C, FTIR (ύ, $cm^{-1}$): 2921-2859 ($CH_2$ stretching), 1740 (C=O), 1599 (C=C), 1473 (C–O Asym), 1419 (N=N), 1224 (C–O Sym). 1H NMR (400 MHz, $CDCl_3$): δ/ppm: 0.85 (t, 3H, $CH_3(CH_2)_7CH_2$ $CH_2$, J = 6.6 Hz), 1.21–1.41 (m, 14H, $CH_3(CH_2)_7$ $CH_2$ $CH_2$), 1.71–1.82 (m, 2H, $CH_3(CH_2)_7CH_2CH_2$), 3.98 (m, 2H, $CH_3(CH_2)_7CH_2CH_2$), 6.94 (m, 2.34 H, Ar–H), 7.19 (s, 1H, Ar–H, E-isomer), 7.42–7.40 (m, 2.10 H, Ar–H), 7.89 (d, 2H, J = 8.2 Hz, Ar–H E-isomer), 8.05–8.14 (m, 3.20 H, Ar–H), 8.2 (d, 0,12 H, J = 8.4 Hz, Ar–H Z-isomer),), 8.4 (d, 0.12 H, J = 8.3 Hz, Ar–H, Z-isomer), 8.6 (d, 1 H, J = 8.5 Hz, Ar–H, E-isomer). Elemental analyses: Found (Calc.): C, 70.61 (70.63); H, 7.64 (7.67); N, 7.25 (7.27); Cl, 6.10 (6.13).

## 3. *Characterizations*

The formation of the supramolecular complexes was confirmed by TA Instruments Co. Q20, differential scanning calorimetry (DSC; USA), polarized-optical microscopy (POM, Wild, Germany).

Calorimetric measurements were carried out using DSC. The instrument was calibrated for temperature, heat and heat flow. Measurements were carried out for small samples (2-3 mg) placed in sealed aluminum pans. All measurements were conducted at a heating rate of 10°C/min in an inert atmosphere of nitrogen gas (10 mL/min). For DSC, the sample was heated from room temperature to 280 °C at heating rate of 10 °C/min under nitrogen atmosphere, and then cooled in the cell to 0 °C. All weighed samples were made using an ultra-microbalance, England, with accuracy ±0.0001.

Transition temperatures for the complexes (**A***6*/**B***n*) were investigated by DSC in

heating and cooling cycles. The types of the mesophase were identified using a standard polarized-optical microscopy POM (Wild, Germany), attached with Mettler FP82HT hot stage. Measurements were made twice and the results were found to have accuracy in transition temperature and enthalpy within ± 0.2.

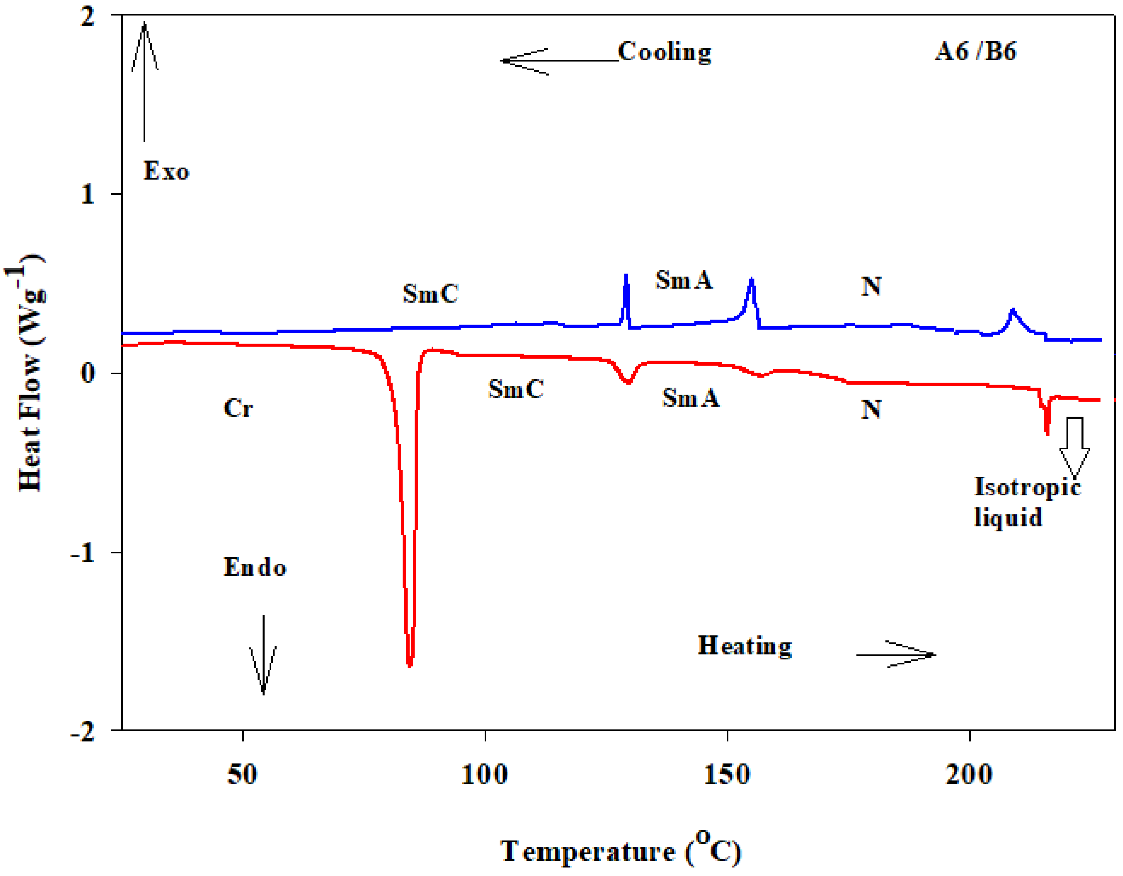


**Figure S3.** DSC thermogram of **A***6*/**B***6* SMHBC at heating rate 10 °C $min^{-1}$during heating and cooling scans.

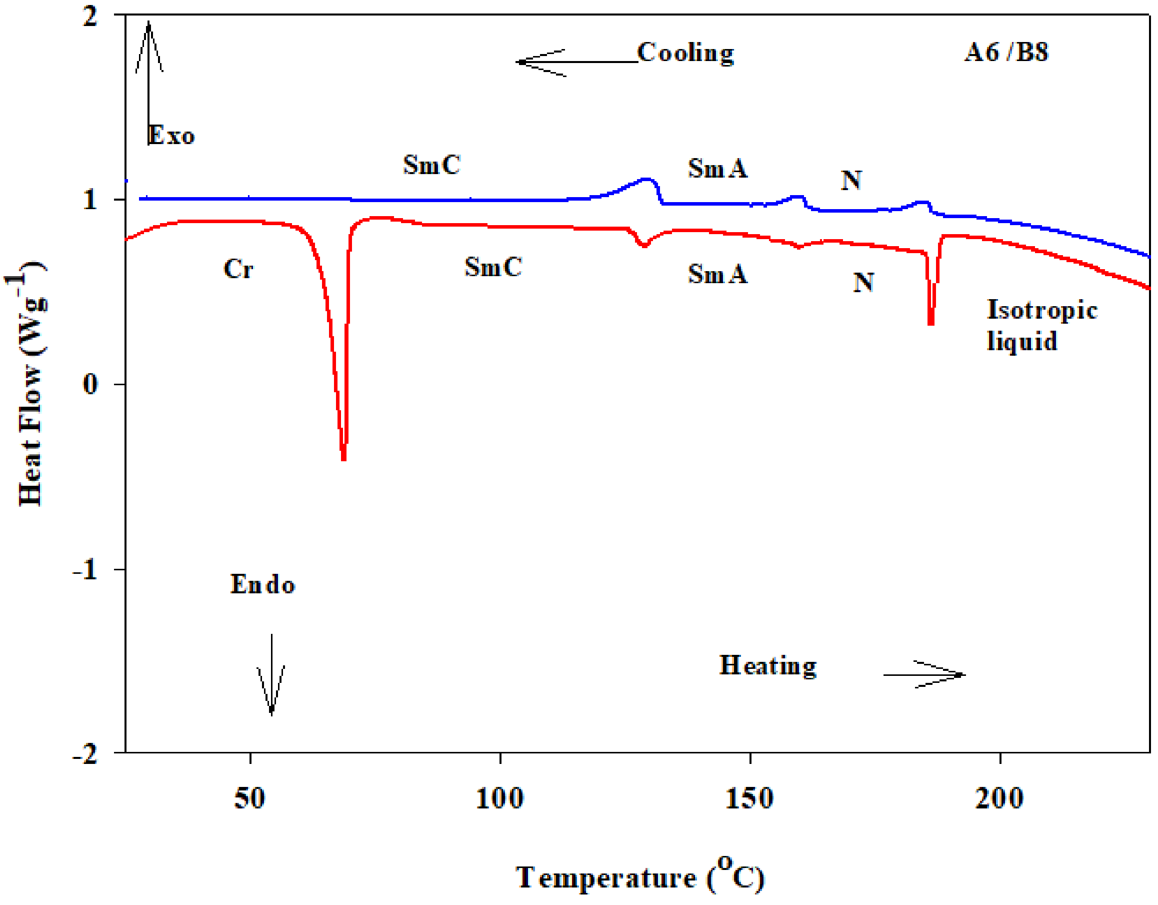


**Figure S4.** DSC thermogram of **A***6*/**B***8* SMHBC at heating rate 10 °C min$^{-1}$during heating and cooling scans.

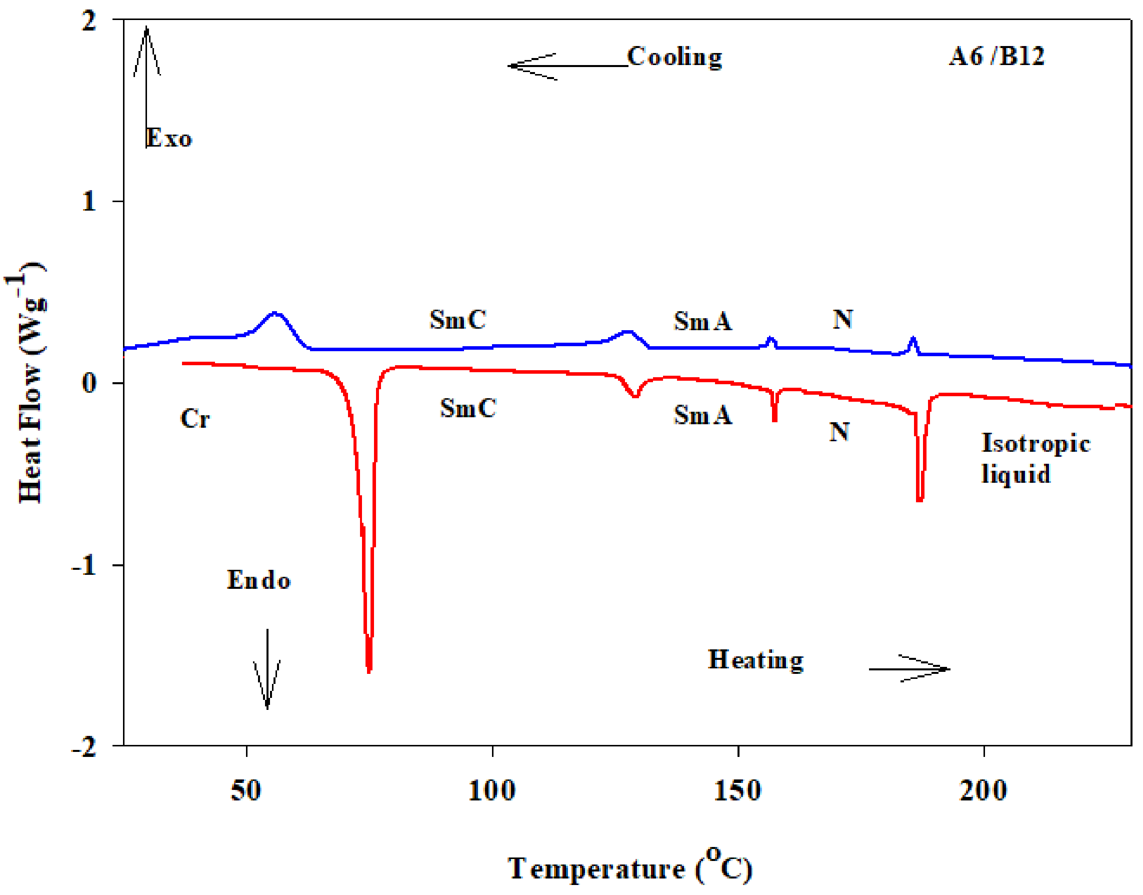


**Figure S5.** DSC thermogram of **A**6/**B**12 SMHBC at heating rate 10 °C min$^{-1}$during heating and cooling scans.

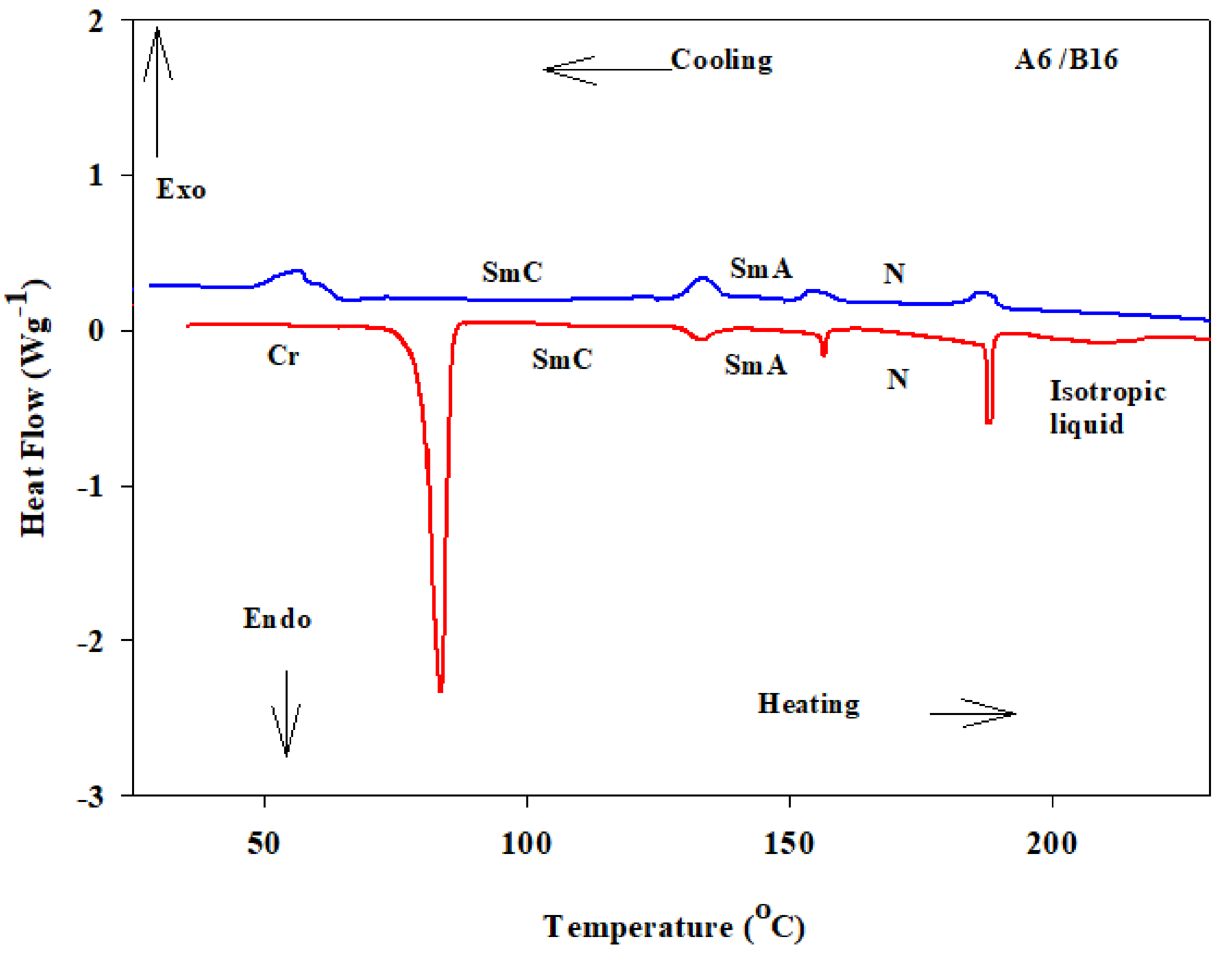


**Figure S6.** DSC thermogram of **A**6/**B**16 SMHBC at heating rate 10 °C min$^{-1}$during heating and cooling scans.
